# Evaluating the utility of an international webinar as a platform to educate students and doctors on the UK core surgical training portfolio

**DOI:** 10.1186/s12909-022-03399-3

**Published:** 2022-04-28

**Authors:** Siddarth Raj, Harroop Bola, Amar Rai, Sarika Grover, Anisha Bandyopadhyay, Vinci Naruka

**Affiliations:** 1 King’s College London Guy’s, King’s, London, UK; 2 St Thomas’ Medical School, London, UK; 3 Imperial College School of Medicine, London, UK; 4 Department of Cardiothoracic Surgery, Imperial College Healthcare NHS Trust, London, UK

**Keywords:** Webinar, Core Surgical Training, Portfolio

## Abstract

**Background:**

Core Surgical Training (CST) is a competitive two-year postgraduate training program in the UK that is scored based on three equally weighted stations: management, clinical and portfolio. Preparing a surgical portfolio can start in medical school, however, there is limited guidance on what forms a competitive portfolio with the majority of advice coming from university resources and national societies which are variable throughout the country. Our aim was to assess the utility of a webinar to educate students and doctors on the CST portfolio to address this disparity.

**Methods:**

Pre- and post-event questionnaires that included demographic data, 10-point Likert scales to self-report confidence on the understanding of the CST portfolio and its domains, and questions on utilising webinars were distributed to attendees. Pre- and post-event responses were paired, and scores were assessed for normality via the Shapiro–Wilk test; the Mann–Whitney U test was used to assess statistical significance. Cohen's d effect sizes were calculated to report standardised differences between pre-and post-event scores.

**Results:**

A total of 177 participants from over 24 countries responded to both questionnaires. A statistically significant improvement in awareness of how to score maximum points was demonstrated across all nine CST domains (*p* < 0.0001). Regardless of whether they were UK-based or international, approximately half of the participants stated a preference for a webinar over an in-person format. Interestingly, most attendees did not feel that their university had provided them with adequate information on preparing for a surgical career with over half of the attendees stating that they had to attend events by external organisations to obtain such information.

**Conclusions:**

This study has demonstrated that a webinar can be effectively utilised to educate students and doctors on the various domains of the CST portfolio and how to maximise points in each section. Such events could address the variability of university resources and national societies across the country and provide equal opportunities for students. Further studies that directly compare webinars with in-person events and investigate long-term outcomes, such as success in CST applications, are required.

**Supplementary Information:**

The online version contains supplementary material available at 10.1186/s12909-022-03399-3.

## Introduction

Core Surgical Training (CST) is a two-year training programme in the United Kingdom (UK) that sits between the foundation programme and higher surgical training. CST is extremely competitive with 2322 applications for only 605 posts in 2020, a competition ratio of 3.84 [1]. Shortlisting for the CST programme is based on self-assessment and evidence verification of the surgical portfolio. This portfolio, alongside the equally weighted 10-min management and clinical stations, make up the scoring system that is used to rank CST applicants.

Creating a competitive application is largely dependent on the preparation of a surgical portfolio, which can begin as early as in medical school. A competitive application is tailored in accordance with the scoring criteria for the CST portfolio, which is divided into nine distinct categories: commitment to speciality; post-graduate degrees, qualifications, and additional degrees; prizes/awards; quality improvement/clinical audit; teaching experience; training in teaching; presentations; publications; and leadership and management [2].

At present, there is limited formal guidance made available to students on what forms a competitive portfolio for CST. The majority of guidance on pursuing a career in surgery in the UK comes from university surgical societies, national societies and mentors, which are variable throughout the country [3]. For example, existing national surgical organisations, such as the Royal College of Surgeons (RCS) and the Association of Surgeons in Training (ASiT), along with medical school surgical societies have previously provided information on how to pursue a career in surgery through in-person seminars, conferences, and events [2, 4]. One way this variability could be addressed is by such organisations delivering webinars on a national, or even international, level that explain how to pursue a career in surgery and how to develop a competitive CST portfolio. This could be done in conjunction with dedicated career talks as it has previously been demonstrated that such events that take place during medical school can serve as an influential factor in the decision to pursue surgery [5, 6].

During the coronavirus disease 2019 (COVID-19) pandemic, access to digital mediums has become increasingly prevalent and has become an efficient modem for students to interact [7–9]. Webinar-based teaching has become the standard throughout medical schools, and it has been noted that this model of teaching is sustainable as it reduces the barriers of participation, cost and time commitment [10]. Therefore, delivering webinars boasts a significant number of advantages by fostering the opportunity to expand exposure to an international audience with minimal costs and administration, while simultaneously improving attendance [6].

Therefore, our main aim was to address the variability in access to information about the CST application and therefore provide information in detail about the CST application in a single digital free webinar format to students based nationally and internationally, from all demographics. This cross-sectional study will report on the utility of a single digital webinar as a platform to improve awareness and understanding of the CST portfolio domains and how to score maximum points in each domain for medical students and professionals.

## Methods

This study was reported in line with the STROBE guidelines, which includes a checklist for cross-sectional studies [11].

### Webinar

A single 90-min free digital webinar session was organised in March 2021. The webinar was designed to educate attendees regarding the CST portfolio and application process. The webinar was designed and delivered by a cardiothoracic surgical trainee in the UK who had prior experience in applying for CST, preparing a CST portfolio, as well as delivering other national webinars. The speaker discussed his own pathway to becoming a surgical trainee, as well as provided an approximate timeline for the application process and provided information on the average number of annually accepted applicants in each deanery of the UK. Most of the webinar then focussed on each domain of the CST application. The speaker offered advice and utilised real-world examples to demonstrate how to score maximum points in each domain. Finally, the speaker offered tips and advice on networking and mentorship. The webinar was conducted over Zoom^Ⓡ^(Zoom Video Communications, USA), a video teleconferencing software that is the most frequently used in medical education [12].

The webinar was open to anyone including pre-medical and current medical students as well as foundation doctors and core trainees. In total, there were 257 attendees at the webinar. Participants voluntarily signed up for this event via an online application form that was advertised through social media platforms, there was no limit on the number of attendees.

### Feedback

A pre-event questionnaire was distributed to attendees to respond to prior to the event (Additional file 1). This questionnaire included demographic questions on sex, medical school, country of origin and stage of medical training. A 10-point Likert scale was used for statements pertaining to participants’ interest in surgery, self-rated awareness of the CST portfolio and each of its domains, and the extent to which their university has provided them with adequate information on how to pursue a career in surgery. “Strongly disagree” was assigned a score of zero and “strongly agree” was assigned a score of 10. This scale was utilised as it effectively enables qualitative information to be quantified for comparison and further statistical analysis.

The post-event questionnaire included 12 of the same questions from the pre-event questionnaire along with feedback on the presenter’s knowledge and ability to communicate (Additional file 2). This questionnaire also included questions on how useful participants found the session, which was also scored using a 10-point Likert scale. Participants were also a handful of questions that focused on comparisons between webinars and in-person events to establish their preferences (Additional file 2). The post-event questionnaire was not piloted before being distributed.

Both pre- and post-webinar questionnaires were hosted on Google Forms (Google, USA) and were anonymised after pre- and post-event responses were paired using Google Sheets (Google, USA). Only participants that filled out both the pre- and post-event questionnaires were eligible for inclusion in this study.

### Statistical methods

The Shapiro–Wilk test was used to assess whether the data was normally distributed. As the data displayed a nonparametric distribution, a Mann–Whitney U test was used to evaluate whether there was a significant difference between pre- and post-event statements. The statistical analysis was performed using GraphPad Prism 9.0.0 (GraphPad Software, La Jolla California, USA). Thereafter, Cohen's d effect size was calculated to report the standardised difference between the pre- and post-event Likert scores.

### Patient and public involvement

No patients were included in this cross-sectional study. All participants in this study provided informed consent for their data to be used anonymously for both research and educational purposes. Participants had the opportunity to opt out of completing either questionnaire at any point. The data collected from both pre- and post-event questionnaires were anonymised prior to data analysis and were stored in password-protected files to comply with General Data Protection Regulation (GDPR).

## Results

A total of 177 attendees completed both the pre- and post-event surveys, 59.9% of which were female. Although participants from 24 countries attended, the majority were predominantly from the UK and were either pre-clinical or clinical medical students (Table 1).

**Table 1:** Baseline characteristics of attendees

Characteristics	Number of participants (%) *N* = 177
**Sex**	
Female	106 (59.9%)
Male	70 (39.5%)
Prefer not to say	1 (0.6%)
**Stage of Training**	
Pre-medical student	3 (1.7%)
Pre-clinical medical student	55 (31.1%)
Clinical medical student	51 (28.8%)
Intercalating	8 (4.5%)
Foundation Doctor	39 (22.0%)
Core Trainee	5 (2.82%)
Speciality Trainee	3 (1.7%)
Physician Associate Student	1 (0.6%)
Other	12 (6.8%)
**Region of origin**	
United Kingdom	112 (63.3%)
Asia	
India	23 (13.0%)
Pakistan	8 (4.5%)
Bangladesh	1 (0.6%)
China	1 (0.6%)
Malaysia	1 (0.6%)
Nepal	1 (0.6%)
Philippines	1 (0.6%)
Sri-Lanka	1 (0.6%)
Europe	
Georgia	5 (2.8%)
Armenia	3 (1.7%)
Czech Republic	3 (1.7%)
Bulgaria	2 (1.1%)
Latvia	2 (1.1%)
Ireland	1 (0.6%)
Portugal	1 (0.6%)
Ukraine	1 (0.6%)
Africa and Middle East	
Jordan	4 (2.3%)
Sudan	2 (1.1%)
Egypt	1 (0.6%)
Iraq	1 (0.6%)
Nigeria	1 (0.6%)
Saudi Arabia	1 (0.6%)
South America	
Colombia	1 (0.6%)

**Fig. 1:**
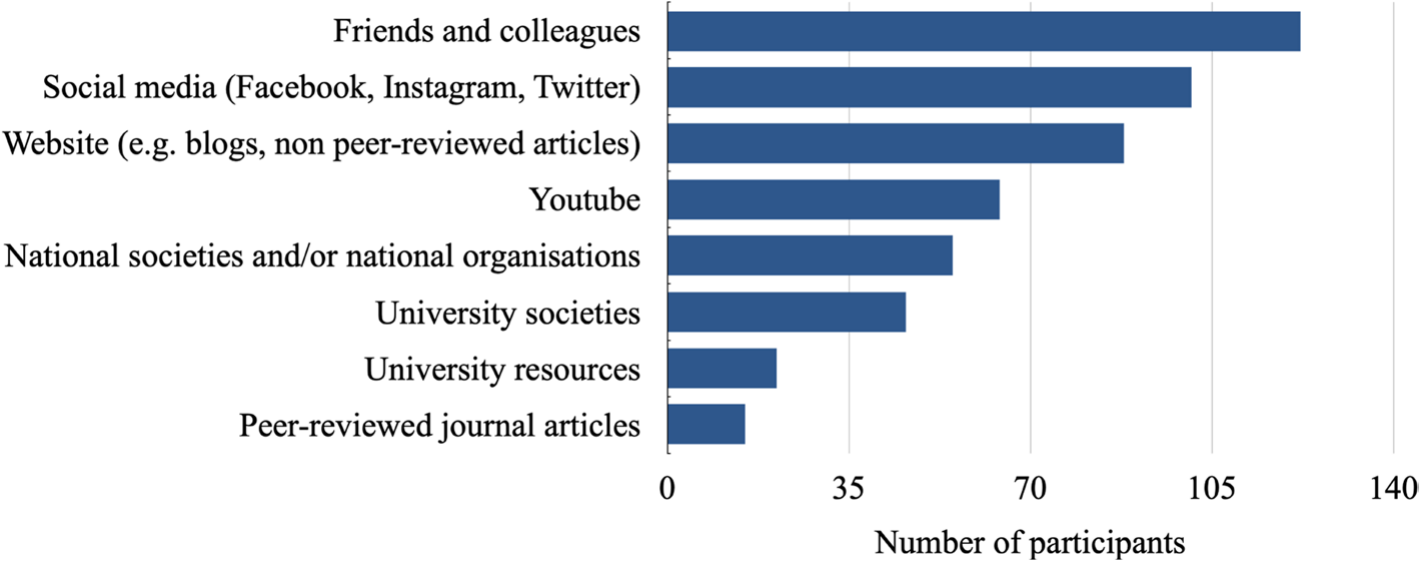
Resources used by attendees to learn about the Core Surgical Training application process. Attendees were allowed to select multiple resources

Only 51 (28.8%) attendees stated that they were aware of the “2021 Core Surgical Training Self-Assessment Scoring Guidance for Candidates” document and how it is assessed. When asked which resources attendees have used to learn about the CST application process, 122 (68.9%) attendees stated that they referred to ‘friends and colleagues,’ followed by ‘social media’ and ‘websites (e.g. blogs, non-peer-reviewed articles),’ which were selected by 101 (57.1%) and 88 (49.7%) attendees, respectively. In comparison, only 55 (31.1%), 46 (26.0%) and 21 (11.9%) students stated they utilised ‘national societies and/or national organisations’, ‘university societies’ and ‘university resources’, respectively to learn about the CST application (Fig. 1).

Most attendees (75.1%) did not agree that their university had provided them with adequate information on how to prepare for a career in surgery with 106 (59.9%) attendees agreeing that they felt they had to attend events organised by external societies or organisations to understand how to prepare for a career in surgery. Furthermore, prior to the event, only 24 (13.6%) attendees strongly agreed that they were aware of what the 2021 CST document entailed and how they could score maximum points overall. After the event, this number increased to 132 (74.6%, *p* < 0.0001). The pre- and post-event median scores for participants’ awareness of each domain of the CST portfolio and how to maximise points have been listed in Table 2; a statistically significant improvement in awareness was demonstrated across all nine domains (*p* < 0.0001).

**Table 2:** Effect of the webinar on questionnaire statements using a 0–10 Likert Scale with *p*-values and Cohen’s d effect sizes

Domains	Pre-event median	Post-event median	*p*-value	Effect size
Interest in pursuing a career in surgery	9	9	0.0591	0.29764
Awareness of the '2021 Core Surgical Training Self-Assessment Scoring Guidance' document and how to potentially score maximum points	3	9	** < 0.0001**	1.75446
Commitment to specialty^a^	3	8	** < 0.0001**	1.60517
Post-graduate degrees and qualifications^a^	4	9	** < 0.0001**	1.63065
Prizes/awards^a^	3	9	** < 0.0001**	1.79296
Quality improvement and clinical audit^a^	4	9	** < 0.0001**	1.65132
Teaching experience^a^	4	9	** < 0.0001**	1.61628
Training in teaching^a^	4	8	** < 0.0001**	1.75193
Presentations^a^	3	8	** < 0.0001**	1.74888
Publications^a^	4	9	** < 0.0001**	1.55544
Leadership and management^a^	4	8	** < 0.0001**	1.62796
Confidence in creating a competitive portfolio for CST	2	8	** < 0.0001**	2.01016

^a^This included awareness of the domain and how to score maximum points

A median score of 10 out of 10 was given from all attendees when asked to rate how knowledgeable the presenter was regarding this subject, how effectively the presenter communicated during the session and how useful the session was overall.

In the post-event questionnaire, 60 (53.6%) UK-based attendees and 28 (43.1%) international attendees declared preference of an online webinar format over an in-person event; only 22 (19.6%) UK-based attendees and 24 (36.9%) international attendees would have preferred an in-person event, the remainder had no preference. Out of all participants, 103 (92.0%) UK-based attendees and 61 (93.8%) international attendees stated that webinars are convenient and make it easier to attend national events such as this. Only 33 (29.5%) UK-based attendees and 21 (32.3%) international attendees stated that webinars limit their ability to network and socialise at events such as these. Out of all the participants, 176 (99.4%) stated that they would both use the tips provided in the session for their own application in the future and recommend this session to others.

Finally, before the event, only 110 (62.1%) strongly agreed that they were interested in pursuing a career in surgery in comparison to 131 (74.0%) of attendees that strongly agreed after the event, however, this difference was statistically insignificant (*p* = 0.0591).

## Discussion

In summary, the use of a single digital webinar showed a statistically significant improvement in participants’ understanding of each of the CST portfolio domains and how to score maximum points on each (Table 2). This should be taken in the context of this webinar having been delivered by a presenter that was deemed to be knowledgeable and able to communicate this information effectively. A statistically significant improvement in participants’ confidence for creating a competitive portfolio was also noted.

This was in keeping with the literature as delivery webinars over digital format have previously exhibited an improvement in participant knowledge and confidence in different subjects [8].

For example, a study by Fereydooni et al. evaluated the use of a single 65-min-long webinar in the improvement of knowledge and preparedness of medical students for vascular surgical training [6]. This study involved testing medical students’ knowledge before and after the event through questions such as “duration of vascular surgery residency training” and “minimum number of applications recommended for a successful match into vascular surgery residency training” with significant improvements observed in the post-event responses (*p* < 0.001), which supports the motion that webinars are an effective method of knowledge translation to students [13].

Moreover, our finding that participants’ self-reported confidence in creating a competitive portfolio significantly improved after an online webinar coincides with the findings by Serebrakian et al., which assessed the impact of a webinar on participants’ knowledge of changes to the plastic surgery residency application process [14]. Residents were reported feeling more confident in matching to the plastic surgery residency programme after the webinar compared to before (*p* < 0.001) alongside medical students after the programme. This supports the use of webinars to improve participant confidence in the delivered topics and as a useful method of information participants on training pathways [14]. Another notable example by Khajuria et al., evaluates the use of a two-day national course in the education of UK medical students of the academic foundation programme (AFP) and it was reported that participants showed a statistically significant improvement in their perceived knowledge, understanding of the application process, confidence, and preparedness in applying for the AFP [9]. This indicates that webinars could potentially serve as an alternative platform to educate participants on postgraduate medical and surgical programmes, however, further research comparing webinars and in-person events are required.

Our results showed that not only UK-based students, but also international students can benefit from digital webinars due to convenience and ease of attendance. This is in keeping with a study by Knipfer et al. that demonstrated attendees’ preference for webinar use for educational purposes, which found that international audiences preferred this format due to the reduction of time consumed and the cost of travelling to other locations [15]; this also indicates that such webinars provide a platform to reach out to a wider audience nationally or internationally. Webinars can also provide the benefit of being recorded for future use and learning [16].

Our study found that more participants utilised ‘friends/colleagues’, ‘social media’ or ‘websites’ to learn about the CST application process as opposed to university resources, and 75.1% of attendees did not agree that their university had provided them with sufficient information regarding a surgical career. A reason for this could be due to the discrepancies in surgical specialty teaching across UK medical schools which can be further supported by the literature [17–19]. For example, Soh et al. reviewed the exposure of oral maxillofacial surgery in the UK undergraduate programme and observed that 64.0–81.9% of medical students reported no exposure during training [17]. This is in contrast with medical students’ exposure to other specialities, such as trauma and orthopaedic surgery, where 80.7% of students undertook a trauma and orthopaedics undergraduate placement as per Malik-Tabassum et al. [18]. In addition, Mayer et al. evaluated the extent to which UK medical schools’ learning outcomes correlated with the ear, nose, and throat (ENT) undergraduate curriculum and found that most medical schools met the learning outcomes for ENT anatomy and physiology, but only a small proportion of medical schools met the outcomes for ENT surgical procedures [19].

Prior to the COVID-19 pandemic, medical students were often exposed to teaching regarding surgical training and CST applications during undergraduate in-person events hosted by student-run surgical societies, which still resulted in foundation doctors feeling neither confident nor diffident about the CST application process [20]. Our study also demonstrated that participants did not tend to learn about CST applications from student-run university societies or even larger national societies and organisations. A study by Patel et al. demonstrated that prior to the COVID-19 pandemic national societies and organisations previously used more traditional teaching methods, including face-to-face seminars and conferences to educate students regarding CST applications, which are less accessible to the wider national and international population and could therefore be a less popular resource [8]. However, given the COVID-19 pandemic, Patel et al. postulate that there will be an increased demand for high quality, engaging and informative webinars [8].

This collectively highlights potential reasons why utilising university, or national resources in the past have not been as popular. It is clear that medical schools vary in terms of exposure to surgery and providing information regarding different surgical specialties. University or national societies and/or organisations previously delivered predominantly in-person events for education on surgical training, therefore potentially reducing accessibility to interested students. Our present study with 177 participants demonstrates that webinars, as opposed to traditional teaching from national and university societies, can increase accessibility to students nationally and internationally and serve as a useful tool in providing students and doctors with a digital resource of information about surgical training and the CST portfolio.

### Limitations

As a cross-sectional analysis, our study is inherently limited by a small sample size based on the number of attendees at a single webinar. Additionally, despite having a diverse and international study population, we did not perform subgroup analysis on each respective country or region of origin as these populations were smaller in size, thus limiting the power of a potential analysis.

The study may also be affected by self-selection bias as the webinar was predominantly attended by individuals who already had an existing interest in surgery and therefore may have been somewhat familiar with the various CST domains and terms used, thus making it easier for them to improve their understanding.

Similarly, sampling bias could have also affected the nature of responses received on the topic of preference between webinars and in-person events as those who prefer the former are inherently more likely to attend this type of event.

Additionally, the data is susceptible to recency bias as attendees completed the post-event questionnaire immediately after the event, which means that attendees could have overestimated their understanding of the CST portfolio and its respective domains. The outcomes in this study are self-reported and therefore subjective, which limits the conclusions we can draw regarding participants’ knowledge retention. Finally, the scope of the study is narrow, focussing on surgical training in the UK only.

Future studies could focus not only on UK-based surgical training, but also other specialties and their respective portfolios or application processes, including that for further surgical specialty training. Additionally, future studies could utilise knowledge-based data as an outcome along with longer-term outcomes, such as whether participants were successful in their respective CST applications to determine the value of a similar webinar. Finally, more research could also be carried out to directly compare the utility of webinars with in-person events.

## Conclusion

This study has demonstrated that a webinar can be effectively utilised as a tool to educate students and doctors on the various domains of the CST portfolio and how to maximise points in each section. This should be taken in the context of the webinar having been delivered by someone who is knowledgeable and able to effectively communicate this information. The majority of attendees did not feel that their university had provided them with adequate information on preparing for a surgical career, and over half of the attendees stated that they had to attend events by external organisations to obtain such information. Almost half of the participants noted their preference for an online webinar over an in-person event. More than half of all participants stated that they were more likely to ask questions in a webinar. Only less than a third of participants felt that webinars limited their ability to network and socialise at such events. Most attendees strongly agreed that webinars are convenient and make it easier to attend such national events.

Such events could address the variability of university resources and national societies throughout the country and could provide students and doctors with equal opportunities, especially if held as a free event. However, further studies that compare the utility of webinars with in-person events and investigate the impact of such events on long-term outcomes, such as success in CST applications, are required.

## Supplementary Information

**Additional file 1.**

**Additional file 2.**

## Data Availability

The data that support the findings of this study are available on request from the corresponding author.
