# Supplementary material for: Evaluating the utility of an international webinar as a platform to educate students and doctors on the UK core surgical training portfolio
Source: BMC Med Educ. 2022 Apr 28;22:329. doi: 10.1186/s12909-022-03399-3 (PMC9047460; doi:10.1186/s12909-022-03399-3)
Supplement: Supplementary file 1 — Additional file 1. [file 12909_2022_3399_MOESM1_ESM.docx]

**Table 1:** Pre-webinar survey.

| Name | |
| --- | --- |
| Email (to match pre-and post-webinar questionnaires) | |
| Sex | |
| Which Medical School do you/did you attend? Please choose other if you are not currently enrolled at Medical school. | 1. University of Aberdeen School of Medicine and Dentistry  2. Anglia Ruskin University School of Medicine  3. Aston University Medical School  4. Barts and The London School of Medicine and Dentistry  5. University of Birmingham College of Medical and Dental Sciences  6. Brighton and Sussex Medical School  7. University of Bristol Medical School  8. University of Buckingham Medical School  9. University of Cambridge School of Clinical Medicine  10. Cardiff University School of Medicine  11. University of Dundee School of Medicine  12. Edge Hill University Medical School  13. The University of Edinburgh Medical School  14. University of Exeter Medical School  15. University of Glasgow School of Medicine  16. Hull York Medical School  17. Imperial College London Faculty of Medicine  18. Keele University School of Medicine  19. Kent and Medway Medical School  20. King's College London GKT School of Medical Education  21. Lancaster University Medical School  22. University of Leeds School of Medicine  23. University of Leicester Medical School  24. University of Liverpool School of Medicine  25. London School of Hygiene & Tropical Medicine  26. University of Manchester Medical School  27. Newcastle University School of Medical Education  28. Norwich Medical School  29. University of Nottingham School of Medicine  30. University of Nottingham - Lincoln Medical School  31. University of Oxford Medical Sciences Division  32. Plymouth University Peninsula Schools of Medicine and Dentistry  33. Queen's University Belfast School of Medicine  34. University of Sheffield Medical School  35. University of Southampton School of Medicine  36. University of St Andrews School of Medicine  37. St George's, University of London  38. University of Sunderland School of Medicine  39. Swansea University Medical School  40. University of Central Lancashire School of Medicine  41. University College London Medical School  42. University of Warwick Medical School  43.Other |
| If you selected ‘other’ in the previous question, please state where you study or work. | |
| What stage of your medical training are you in currently? | - Pre-medical school - Pre-clinical years in medical school - Intercalating (taking time out medicine to do a BSc, MSc, etc.) - Clinical years in medical school - PA Student - Physician Associate - Foundation Year 1 Doctor - Foundation Year 2 Doctor - Foundation Year 3 Doctor - Core Trainee - Speciality Trainee - Fellow - Consultant - Other: |
| If you are a Junior Doctor in the UK, please choose your Foundation Deanery. If this does not apply to you, please choose 'Not applicable - please select this if you are not a Junior Doctor.' | 1. Not applicable – please select this if you are not a Junior Doctor. 2. East Anglia 3. Essex, Bedfordshire & Hertfordshire (EBH) 4. Leicester, Northamptonshire & Rutland (LNR) 5. North Central and East London 6. North West London 7. North West of England 8. Northern 9. Northern Ireland 10. Oxford 11. Peninsula 12. Scotland 13. Severn 14. South Thames 15. Trent 16. Wales 17. Wessex 18. West Midlands Central 19. West Midlands North 20. West Midlands South 21. Yorkshire & Humber |
| Are you aware of the '2021 Core Surgical Training Self-Assessment Scoring Guidance for Candidates' document AND how it is assessed? | - Yes - No |
| **Prior to attending this event, please rate to what extent you agree with the following statements:** | |
| I am interested in pursuing a career in surgery. | Likert Scale 0-10: Strongly Disagree to Strongly Agree. 5=Neutral. |
| I am aware of what the '2021 Core Surgical Training Self-Assessment Scoring Guidance' document entails AND what I could potentially do to score the maximum number of points overall. | Likert Scale 0-10: Strongly Disagree to Strongly Agree. 5=Neutral. |
| I understand what is classified as 'Commitment to specialty (all surgical specialties)' and I am aware of what I can do to score maximum points in this section. | Likert Scale 0-10: Strongly Disagree to Strongly Agree.  5=Neutral. |
| I understand what is included in the 'Postgraduate degrees and qualifications and additional degrees' section and I am aware of what I can do to score maximum points in this section. | Likert Scale 0-10: Strongly Disagree to Strongly Agree.  5=Neutral. |
| I understand what is classified as 'Prizes/Awards' and I am aware of what I can do to score maximum points in this section. | Likert Scale 0-10: Strongly Disagree to Strongly Agree.  5=Neutral. |
| I understand what a 'Quality Improvement Project' and 'Clinical Audit' are and how I can score maximum points in the 'Quality Improvement/Clinical Audit' section. | Likert Scale 0-10: Strongly Disagree to Strongly Agree.  5=Neutral. |
| I understand what is classified as 'Teaching Experience' and I know how I can score maximum points in this section. | Likert Scale 0-10: Strongly Disagree to Strongly Agree.  5=Neutral. |
| I understand what is classified as 'Training in Teaching' and I know how I can score maximum points in this section. | Likert Scale 0-10: Strongly Disagree to Strongly Agree.  5=Neutral. |
| I understand what 'Presentations' are in the context of CST and I know how I can score maximum points in this section. | Likert Scale 0-10: Strongly Disagree to Strongly Agree.  5=Neutral. |
| I understand what 'Publications' are in the context of CST and I know how I can score maximum points in this section. | Likert Scale 0-10: Strongly Disagree to Strongly Agree.  5=Neutral. |
| I understand what is classified as 'Leadership and Management' and I know how I can score maximum points in this section. | Likert Scale 0-10: Strongly Disagree to Strongly Agree.  5=Neutral. |
| I am confident about how to create a competitive portfolio for Core Surgical Training. | Likert Scale 0-10: Strongly Disagree to Strongly Agree.  5=Neutral. |
| To what extent do you agree with this statement? ‘My university provides/provided adequate information on how to prepare for a career in Surgery’ | Likert Scale 0-10: Strongly Disagree to Strongly Agree.  5=Neutral. |
| To what extent do you agree with the following statement? "I have to attend events organised by external societies or organisations to understand how to prepare for a career in Surgery’ | Likert Scale 0-10: Strongly Disagree to Strongly Agree.  5=Neutral. |
| What resource(s) have you used, if any, to learn about the Core Surgical Training application process? You can select more than one. | - Friends and colleagues - National societies and/or national organizations - Peer-reviewed journal articles - Social Media (e.g. Instagram, Twitter, Facebook) - University resources - University societies - Websites (e.g., blogs, non-peer-reviewed articles) - YouTube - Other: |
