# Supplementary material for: Evaluating the utility of an international webinar as a platform to educate students and doctors on the UK core surgical training portfolio
Source: BMC Med Educ. 2022 Apr 28;22:329. doi: 10.1186/s12909-022-03399-3 (PMC9047460; doi:10.1186/s12909-022-03399-3)
Supplement: Supplementary file 2 — Additional file 2. [file 12909_2022_3399_MOESM2_ESM.docx]

**Table 2:** Post-webinar survey.

| Name | |
| --- | --- |
| Email (to match pre-and post-webinar questionnaires) | |
| **Having attended this event, please rate to what extent you agree with the following statements:** | |
| I am interested in pursuing a career in surgery. | Likert Scale 0-10: Strongly Disagree to Strongly Agree. 5=Neutral. |
| I am aware of what the '2021 Core Surgical Training Self-Assessment Scoring Guidance' document entails AND what I could potentially do to score the maximum number of points overall. | Likert Scale 0-10: Strongly Disagree to Strongly Agree. 5=Neutral. |
| I understand what is classified as 'Commitment to specialty (all surgical specialties)' and I am aware of what I can do to score maximum points in this section. | Likert Scale 0-10: Strongly Disagree to Strongly Agree.  5=Neutral. |
| I understand what is included in the 'Postgraduate degrees and qualifications and additional degrees' section and I am aware of what I can do to score maximum points in this section. | Likert Scale 0-10: Strongly Disagree to Strongly Agree.  5=Neutral. |
| I understand what is classified as 'Prizes/Awards' and I am aware of what I can do to score maximum points in this section. | Likert Scale 0-10: Strongly Disagree to Strongly Agree.  5=Neutral. |
| I understand what a 'Quality Improvement Project' and 'Clinical Audit' are and how I can score maximum points in the 'Quality Improvement/Clinical Audit' section. | Likert Scale 0-10: Strongly Disagree to Strongly Agree.  5=Neutral. |
| I understand what is classified as 'Teaching Experience' and I know how I can score maximum points in this section. | Likert Scale 0-10: Strongly Disagree to Strongly Agree.  5=Neutral. |
| I understand what is classified as 'Training in Teaching' and I know how I can score maximum points in this section. | Likert Scale 0-10: Strongly Disagree to Strongly Agree.  5=Neutral. |
| I understand what 'Presentations' are in the context of CST and I know how I can score maximum points in this section. | Likert Scale 0-10: Strongly Disagree to Strongly Agree.  5=Neutral. |
| I understand what 'Publications' are in the context of CST and I know how I can score maximum points in this section. | Likert Scale 0-10: Strongly Disagree to Strongly Agree.  5=Neutral. |
| I understand what is classified as 'Leadership and Management' and I know how I can score maximum points in this section. | Likert Scale 0-10: Strongly Disagree to Strongly Agree.  5=Neutral. |
| I am confident about how to create a competitive portfolio for Core Surgical Training. | Likert Scale 0-10: Strongly Disagree to Strongly Agree.  5=Neutral. |
| Please rate (out of 10) how knowledgeable the presenter was regarding this subject. | Likert Scale 0-10: Not at all knowledgeable to Extremely knowledgeable. |
| Please rate (out of 10) how effectively the presenter communicated during the session. | Likert Scale 0-10: Not at all effective to Extremely effective. |
| Please rate (out of 10) how useful this session was. | Likert Scale 0-10: Not at all useful to Extremely useful. |
| Have you attended an online webinar on Core Surgical Training and/or Speciality Surgical Training before? | - Yes - No |
| To what extent do you agree with the following statements: Webinars are convenient and make it easy to attend national events like this event. | Likert Scale 0-10: Strongly Disagree to Strongly Agree. 5= Neutral. |
| To what extent do you agree with the following statements: Webinars limit my ability to network and socialise at a national event like this. | Likert Scale 0-10: Strongly Disagree to Strongly Agree. 5= Neutral. |
| If there were no restrictions on social-distancing, which format would you prefer for this type of event? | - Face-to-face, in-person event - Online webinar - Neutral |
| Are you more likely to ask questions at a face-to-face in-person event or at an online webinar? | - Face-to-face, in-person event - Online webinar - Neutral (equally likely) |
| Will you use the tips provided in this session for your own application in the future? | - Yes - No |
| Would you recommend this session to others? | - Yes - No |
